# Providers’ Knowledge and Perceptions of Bariatric Surgery: a Systematic Review

**DOI:** 10.1007/s11695-023-06827-5

**Published:** 2023-09-23

**Authors:** Nithya D. Rajeev, Jamil S. Samaan, Agnes Premkumar, Erin Yu, Nitin Srinivasan, Kamran Samakar

**Affiliations:** 1 Division of Upper GI and General Surgery, Department of Surgery, Keck School of Medicine, University of Southern California, 1510 San Pablo St., Suite 514, Los Angeles, CA 90033 USA; 2 Department of Medicine, Karsh Division of Gastroenterology and Hepatology, Cedars-Sinai Medical Center, 8700 Beverly Blvd, Los Angeles, CA 90048 USA; 3 Department of General Surgery, Creighton University of Phoenix, 3100 N. Central Ave, Phoenix, AZ 85012 USA

**Keywords:** Bariatric surgery, Perception, Provider knowledge, Physician knowledge, Bariatric surgery utilization

## Abstract

Bariatric surgery remains underutilized despite its proven efficacy in the management of obesity. Provider perceptions of bariatric surgery are important to consider when discussing utilization rates. PubMed, SCOPUS, and OVID databases were searched in April 2023, and 40 published studies discussing providers’ knowledge and perceptions of bariatric surgery were included. There were generally positive perceptions of the efficacy of bariatric surgery, although overestimations of surgical risks and postoperative complications were common. Providers’ previous training was associated with knowledge and perceptions of bariatric surgery and familiarity with perioperative management across studies. These perceptions were also associated with referral rates, suggesting that inadequate provider knowledge may contribute to bariatric surgery underutilization. We advocate for increased bariatric surgery-related education throughout all stages of medical training and across specialties.

## Introduction

Obesity is a public health crisis and a notable risk factor for numerous diseases, including cardiovascular disease, diabetes, osteoarthritis, Alzheimer’s, depression, and malignancies [1].The global prevalence of obesity has nearly tripled in the past 40 years, with over 1 billion people now meeting criteria for obesity [2]. Within the United States (US), the prevalence among adults increased from 36 to 41.9% from 2011–2014 to 2017–2020, respectively [3, 4]. Bariatric surgery has been shown to result in sustained long-term weight loss, lower morbidity and mortality, and significant improvements in obesity related comorbidities [5–7].

Despite the literature demonstrating its efficacy, fewer than 1% of eligible candidates worldwide undergo bariatric surgery [8]. This underutilization is likely in part due to healthcare access, and other economic, psychosocial, and systemic factors. Studies have previously shown negative and unrealistic perceptions of bariatric surgery among patients and the general public that may lead to underutilization [9]. Furthermore, previous literature has explored referral patterns for bariatric surgery and identified provider familiarity with bariatric surgery as a possible barrier [10].

Healthcare providers other than bariatric surgeons have an integral role in caring for patients affected by obesity regarding education, treatment options, and perioperative care. We conducted a comprehensive, up-to-date, systematic review of the literature investigating the perceptions and familiarity of healthcare providers with the role of bariatric surgery in the treatment of obesity. We highlight provider knowledge of bariatric surgery, reported confidence in providing perioperative care as well as perceptions regarding its safety and efficacy.

## Methods

**Fig. 1:**
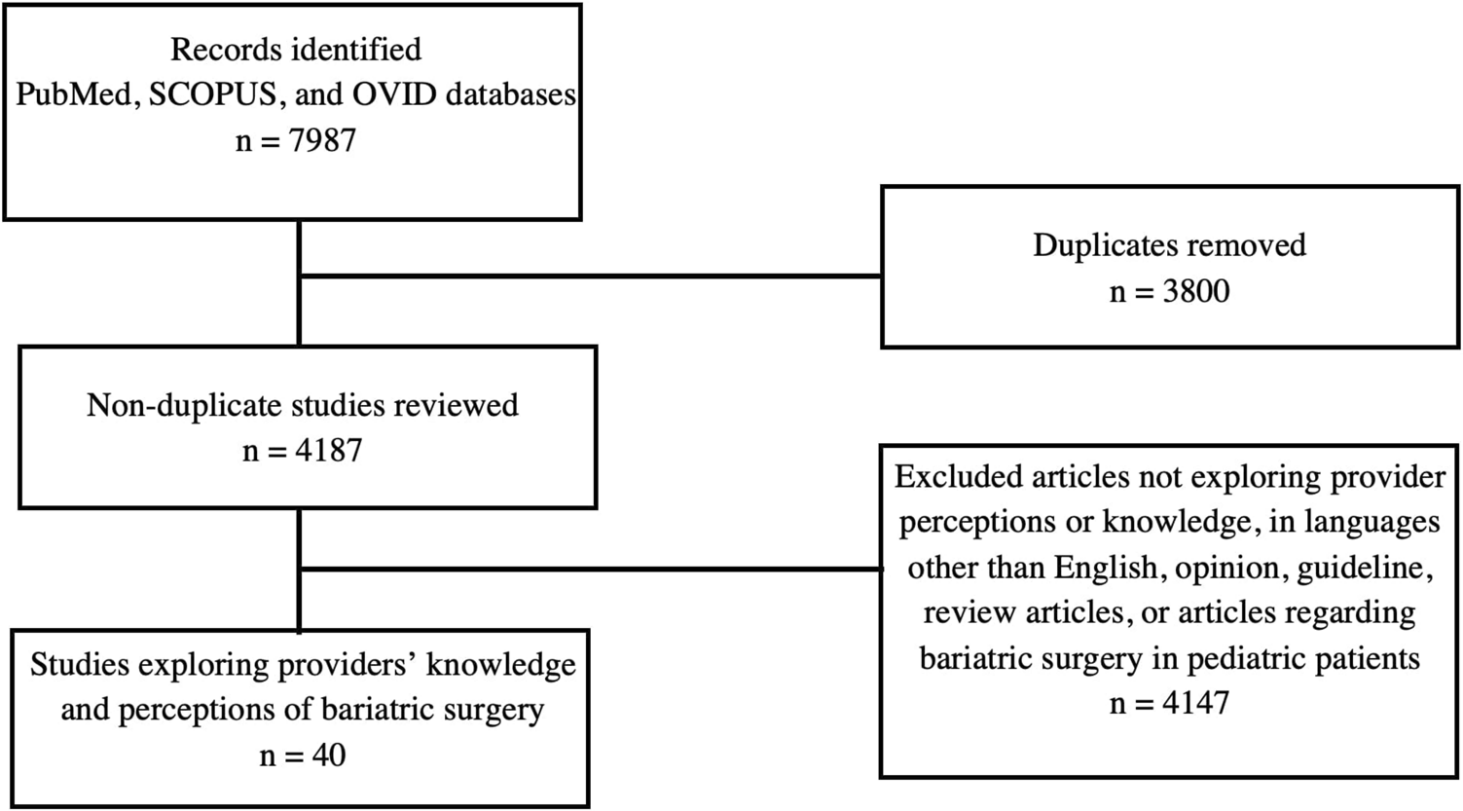
Summary of literature search including inclusion and exclusion criteria

Per the PICO framework, among healthcare providers, we sought to (1) describe knowledge and perceptions of bariatric surgery, (2) investigate which factors are associated with knowledge and perceptions, and (3) investigate the impact of these perceptions on the extent and quality of care provided. Guidelines outlined in the Preferred Reporting Items for Systematic Reviews and Meta-Analyses (PRISMA) were used in developing this systematic review. PubMed, SCOPUS, and OVID were searched in April 2023 with the terms “bariatric surgery perceptions,” “bariatric surgery physician perceptions,” “bariatric surgery provider perceptions,” “bariatric surgery impressions,” “bariatric surgery physician impressions,” “bariatric surgery provider impressions,” “bariatric surgery attitudes,” “bariatric surgery physician attitudes,” “bariatric surgery provider attitudes,” “bariatric surgery knowledge,” “bariatric surgery physician knowledge,” and “bariatric surgery provider knowledge.” Handsearching of citations within included studies was also conducted. A total of 7987 articles were identified and 3800 duplicates were removed, leaving 4187 articles for preliminary screening of titles and abstracts. Inclusion criteria included studies that examined provider knowledge, attitudes, impressions, or perceptions of bariatric surgery. Providers were defined as primary care providers, specialists, and allied health professionals. Exclusion criteria included non-English articles, review articles, opinion articles, guidelines, and articles about perceptions of bariatric surgery in pediatric populations. After a comprehensive screening of titles and in-article citations by two independent reviewers (Fig. 1), an abstract review was conducted for 69 articles, a full-text review was conducted for 54 studies by two reviewers, and 40 studies were included (Table 1). Outcomes were organized into several categories, including knowledge of eligibility and procedure options (Table 2), perceptions of safety and efficacy (Table 3), and factors associated with initiating discussions about bariatric surgery and providing perioperative care (Table 4).

**Table 1:** Summary of characteristics and design for studies included in this systematic review

Author	Location	Study period	Study design	Sample size (*n*)	Response rate	NOS
Thuan et al., 2005	France	NR	• Survey sent to 744 general practitioners registered by the regional representation of the Health Department	607	81.6%	5
Avidor et al., 2007	USA	Apr 2004–Nov 2004	• Questionnaires self-administered at 6 national meetings to physicians of 6 specialty areas: BARI, OBGYN, IM, ENDO, CARD, and FP	478	NR	6
Sansone et al., 2007	USA	NR	• Survey mailed to 246 physicians from the department of IM, FM, and OBGYN in a community hospital	99	40%	4
Balduf et al., 2008	NC, USA	NR	• Survey mailed to 400 FP and 400 IM physicians from North Carolina Health Professions Data System	288	47%	8
Ferrante et al., 2009	NJ, USA	March 2008	• Survey of family physicians who were members of the New Jersey Family Medicine Research Network and 136 additional family physicians from a Blue Cross Blue Shield provider directory	255	53%	7
Salinas et al., 2011	USA	2009	• Questionnaire sent to 1000 family physicians and general internists from a national database of “opted-in” physicians matched to AMA Physician Masterfile	293	NR	6
Sarwer et al., 2012	Philadelphia, PA, USA	NR	• Survey e-mails sent to physicians affiliated with an academic medical center and community-based physicians identified through databases	93	27.4%	7
Claridge et al., 2014	Wellington, New Zealand	NR	• Qualitative study involving series of 12 semi-structured interviews with general practitioners	12	NR	3
Giaro et al., 2014	Poland	2010–2011	• Anonymous questionnaire administered to surgeons during local educational conferences	143	NR	4
Glauser et al., 2015	USA	Feb 2013	• Case vignette survey sent to 1625 PCPs, ENDOs, CARDs, and BARIs identified through Annual AMA Physician Characteristics and Distribution US Report	300	18.46%	6
Kim et al., 2015	New South Wales, Australia	Nov 2013–July 2014	• Semi-structured interviews among general practitioners working in primary care organizations	24	75%	6
Stanford et al., 2015	USA	July 2014 – Oct 2014	• Online survey sent to PCPs affiliated with Massachusetts General Hospital	76	32%	6
Tork et al., 2015	Cincinnati, OH, USA	NR	• Survey administered to PCPs affiliated with The Good Samaritan TriHealth Hospital	57	35.4%	5
Auspitz et al., 2016	Canada	2014	• Survey administered to all physicians practicing FM and listed in the Canadian Medical Directory	165	12%	5
Funk et al., 2016	WI, USA	NR	• Focus groups conducted with PCPs who were members of the Wisconsin Research and Education Network	16	61.5%	5
Hirpara et al., 2016	Canada	Sep 2012–Dec 2012	• Survey administered to attendees of two general surgery conferences, and recipients of the electronic newsletters from OAGS and CAGS	167	10%	8
Jung et al., 2016	Germany	NR	• Questionnaire distributed by mail to general practitioners and internists found in the telephone directory	201	16.3%	5
Major et al., 2016	Poland	2015–2016	• Prospective anonymous questionnaire-based study distributed to GPs, physicians from IM, radiology, ophthalmology, endocrinology, palliative care, occupational medicine, public health, and geriatrics	200	NR	4
Stolberg et al., 2017	Denmark	Feb 2016–Apr 2016	• Survey administered to 300 PCPs systematically selected from a health platform from the Danish Ministry of Health and Danish Regions	133	44%	7
Zacharoulis et al., 2017	Thessaly, Greece	NR	• Self-administered survey distributed to 500 physicians, endocrinologists, cardiologists, pulmonologists, gastroenterologists, orthopedics, gynecologists, general surgeons, and other specialties	300	60%	6
Falvo et al., 2018	PA, USA	NR	• Survey emailed to 160 PCPs at Allegheny Health Network	45	28.1%	5
Martini et al., 2018	France	May 2017–July 2017	• E-questionnaire developed by two bariatric surgeons distributed to practicing general practitioners and FPs	288	12.9%	7
McGlone et al., 2018	England	2015	• Survey administered to three different PCP consortiums	35	9.16%	5
Simon et al., 2018	MI, USA	May 2017	• Survey administered to providers in primary care (IM, FM, and women’s health) and endocrinology in a large health system	111	26%	4
Conaty et al., 2020	Evanston, IL, USA	Mar 2018–Jun 2018	• Survey administered to PCPs at the Northshore University Health System	150	28%	4
El-Beheiry et al., 2020	Manitoba, Canada	July 2015–Sep 2015	• Questionnaire mailed to all registered FPs and attendees of the 2016 provincial conference of FPs	131	13.1%	6
Elliott et al., 2020	Denmark	NR	• Questionnaires administered to orthopedic surgeons, obstetricians/gynecologists, 300 endocrinologists, 169 physicians treating patients with obstructive sleep apnea	345	44%	4
Lopez et al., 2020	WI, USA	NR	• Questionnaire emailed to PCPs at the Medical College of Wisconsin	41	33.9%	6
Egerer et al., 2021	Aachen, Germany	Oct 2019–Mar 2020	• Survey administered to PCPs in the region of a bariatric surgery center at the University Hospital	204	31%	6
Memarian et al., 2021	Skåne & Kronoberg, South Sweden	2019	• Survey emailed to all PCPs with available email addresses	157	14%	6
Özgüc et al., 2021	Turkey	2019	• Survey distributed to PCPs through social media, association websites, and randomly selected physicians	1044	NR	4
Sbraccia et al., 2021	Italy	Jun–July 2018	• Online survey administered to persons with obesity and healthcare professionals from Italian cohort of ACTION-IO study	302 (HCPs)	NR	6
Zevin et al., 2021	Ontario, Canada	Oct 2017–Jun 2018	• Survey emailed to PCPs practicing in the Southeast Local Health Integration Network	92	15.6%	6
Alenezi et al. 2022	Saudi Arabia	Mar 2022–Aug 2022	• Study conducted among PCPs in northern Saudi Arabia who work in public hospital settings	280	NR	8
Carrasco et al., 2022	Sweden	2021	• Questionnaire survey sent to PCPs to assess knowledge, attitudes, and adherence to guidelines for obesity treatment guidelines	235	14.3%	3
Holmes et al., 2022	Ontario, Canada	2020	• Survey emailed to all ENDOs, endocrinology residents, and diabetes nurse practitioners at the University of Toronto	48	NR	5
Murtha et al., 2022	USA	Aug 2016–May 2017	• Interview-based study conducted among eligible patients and providers (PCPs, bariatric surgeons, registered dieticians, and health psychologists)	40 (providers)	22%	4
Ouni et al., 2022	USA	July 2021–Aug 2021	• Questionnaire administered to PCPs at the Mayo Clinic healthcare system	130	40%	4
Zawadzka et al., 2022	Poland	Oct 2019 and Jan 2021	• Survey administered to internists, diabetologists, and trainees in both fields attending two virtual conferences via an online, anonymous and self-administered format	64	37.9%	7
Mojkowska et al., 2023	Poland	Jan 2019–Sep 2020	• Survey conducted among active HCPs (physicians, nurses, physiotherapists, paramedics)	184	NR	4

Abbreviations: *ACTION-IO* Awareness, Care & Treatment in Obesity Management–An International Observation, *AMA* American Medical Association, *BARI* bariatric medicine, *CAGS* Canadian Association of General Surgeons, *CARD* cardiologists, *ENDO* endocrinologists, *FM* family medicine, *FP* family practitioners, *HCP* healthcare professional, *IM* internal medicine, *NOS* Newcastle–Ottawa Scale, *NR* not reported, *OAGS* Ontario Association of General Surgeons, *OBGYN* obstetrics/gynecology, *PCP* primary care physician

**Table 2:** Knowledge of eligibility and procedure types for surgical management of obesity

Author	Routinely weigh patients	Knowledge about eligibility criteria and indications for BSY	Knowledge about BSY procedure options	Statistical test
Avidor et al., 2007		• Average degree of familiarity with NIH eligibility guidelines (range 1–5; 1 = not familiar, 5 = very familiar): ○ BARI (3.9), OBGYN (2.3), IM (2.4), ENDO (3.3), CARD (2.5), FM (3)	• Average degree of familiarity: ○ All physicians: RYGB (3.2), LRYGB (3.1), LAGB (3.0) ○ BARI: RYGB (3.9), LRYGB (3.9), LAGB (2.9) ○ OBGYN: RYGB (2.9), LRYGB (2.7), LAGB (2.5) ○ IM: RYGB (3.2), LRYGB (2.9), LAGB (2.6) ○ ENDO: RYGB (3.5), LRYGB (3.6), LAGB (3.6) ○ CARD: RYGB (2.7), LRYGB (2.5), LAGB (2.6) ○ FM: RYGB (3.3), LRYGB (3.1), LAGB (3.8)	• 5-point Likert Scale • Descriptive statistics
Balduf et al., 2008		• Percentage who read the NIH 2000 guidelines ○ Referring (14.7%), non-referring (3%) (*p* = 0.02)	• Average score from 10-question assessment of general knowledge ○ Referring (6.9 ± 1.4), non-referring (6.2 ± 1.8), (*p* = 0.006)	• Descriptive statistics
Ferrante et al., 2009			• Knows “much” or “very much” about surgical interventions for obesity: 44%	• Descriptive statistics
Salinas et al., 2011		• Would refer patient with BMI > 40 kg/m^2^ with comorbidities for BSY as a next step in management: 17.4% agree ○ 15.5% FP, 19.3% IM		• Descriptive statistics
Giaro et al., 2014		• Correctly answered question about indications: 77% • Able to use knowledge in practice: 53.1%	• Correct knowledge concerning surgical methods applied in treatment of obesity: 92.3% • Assessment of knowledge: ○ 11.9% completely satisfactory ○ 21.7% insufficient but I study it myself ○ 58.7% inadequate, interested in learning more ○ 7.7% minor, but I do not need it in my practice	• Descriptive statistics
Glauser et al., 2015		• Mean familiarity scores (10 point scale): ○ USPSTF screening and management of obesity in adults: BARI (5.8), CARD (3.5), ENDO (5), PCP (5.6) ○ NHLBI guidelines for management of obesity: BARI (5.2), CARD (3.5), ENDO (5), PCP (4.6) ○ AACE/TOS/ASMBS guidelines for perioperative nutritional, metabolic, and nonsurgical support of BSY patients: BARI (6.5), CARD (2.8), ENDO (6.2), PCP (4) ○ ISCI guidelines of obesity: BARI (3.7), CARD (2.5), ENDO (3.2), PCP (3.7)	• Correctly identified evidence-based excess weight loss for patient who is 150 pounds overweight 1 year after laparoscopic RYGB ○ 40% bariatricians, 20% cardiologists, 38% endocrinologists, 14% PCPs	• Descriptive Statistics
Stanford et al., 2015		• Correctly identified qualifying BMI: No obesity related training (51.7%), previous obesity related training (65.9%)	• Familiar with average expected excess body weight loss from RYGB: No obesity related training (27%), previous obesity related training (73%) • Familiar with the national 30 day mortality rate of RYGB: No obesity related training (32%), previous obesity related training (68%) • Correctly identified postoperative time frame within with patients are expected to achieve maximum weight loss: No obesity related training (43%), previous obesity related training (57%) • Correctly answered more than half of knowledge questions: No obesity related training (46%), previous obesity related training (54%), *p* < 0.05 • Younger PCPs (age 20–39) more likely to have obesity training than PCPs aged 40–49 or 50 + (OR: 0.08, 95% CI: 0.008–0.822 and OR: 0.03, 0.004–0.321, respectively)	• Descriptive statistics • Chi-square test
Tork et al., 2015		• Not familiar with indications: 46% disagree, 19% strongly disagree • BMI > 35 kg/${\mathrm{m}}^{2}$ and comorbidities related to obesity are an indication to refer patient: 85% strongly agree/agree		• Descriptive statistics • 5-point Likert scale
Auspitz et al., 2016	• Every visit: 10.7% • Every year: 80.7%		• Correct identification of 30-day mortality rate: LRYGB (11.2%), LSG (12.6%), LAGB (28.9%) • Correct identification of 30-day morbidity rate: LRYGB (13%), LSG (14.3%), LAGB (30.6%) • “I feel comfortable explaining the procedural options to a patient”: All FPs (23.8%) ○ Previously referred (33.9%), non-referring (5.6%) (*p* = 0.013)	• Descriptive statistics
Funk et al., 2016			• Most PCPs were not sure which BSY approach, open vs. laparoscopic, was performed routinely • Limited familiarity with laparoscopic vertical sleeve gastrectomy ○ Most commonly performed BSY type in the US	• Qualitative
Hirpara et al., 2016	• All surgeons (33%), BS (75%), non-BS (47.6%), *p* = 0.039	• Correctly identify NIH eligibility criteria: All Surgeons (36.3%), ○ BS (85%), non-BS (46.9%) (*p* = 0.002)	• Correctly identify 30-day mortality risk: ○ LRYGB: All surgeons (22.2%), BS (45%), non-BS (16.5%) (*p* = 0.006) ○ LSG: All surgeons (14.3%), BS (70%), non-BS (10.3%) *(p* = 0.024) ○ LAGB: All surgeons (48%), BS (90%), non-BS (37.2%) (*p* < 0.001) • Correctly identify 30-day morbidity: ○ LRYGB: All surgeons (32.7%), BS (35%), non-BS (32.1%) (p = 0.546) ○ LSG: All surgeons (18.6%), BS (20%), non-BS (18.2%) (*p* = 0.314) ○ LAGB: All surgeons (35.1%), BS (40%), non-BS (33.8%) (*p* = 0.241)	• Descriptive Statistics • Pearson Chi-Square Test
Jung et al., 2016			• Rated their knowledge as moderate to good: > 70%	• Linear regression • Logistic regression
Major et al., 2016		• Knows the indications: 81.8%	• Can explain to their patient how the most common procedures are conducted: 75.5% • Can name the most popular procedure: 69.8%	• Descriptive Statistics
Stolberg et al., 2017		• Have good knowledge of national referral criteria: 70% agree/strongly agree		• Descriptive statistics • 5-point Likert Scale
Zacharoulis et al., 2017			• Level of familiarity with various procedure types: ○ Intragastric balloon: 31.7% not at all, 56.7% a little, 11.7% a lot ○ Adjustable gastric banding: 28.7% not at all, 57.7% a little, 13.7% a lot ○ Laparoscopic sleeve gastrectomy: 40.3% not at all, 49.0% a little, 10.7% a lot ○ Roux-en-Y gastric bypass: 56.0% not at all, 38.7% a little, 5.3% a lot ○ Mini-gastric bypass: 63.3% not at all, 32.0% a little, 1.7% a lot ○ Biliopancreatic diversion with or without duodenal switch: 73.7% not at all, 25.3% a little, 1.0% a lot	• Descriptive Statistics
Falvo et al., 2018	• “Always” calculate BMI: 88.9%	• Correctly identified > 2 eligibility criteria: 57.1%	• Above average knowledge of obesity in respective region vs national average: 74.4% • Correctly identified medical problems that can be improved by BSY: 66.7%	• Descriptive Statistics
Martini et al., 2018	• Measure weight “each visit”: 74.3%	• Aware of national guidelines for bariatric surgery: 32.3%	• Familiarity with surgical procedures: ○ Gastric banding: 87.9% ○ Sleeve gastrectomy: 92%	• Descriptive Statistics
McGlone et al., 2018			• Median estimated early mortality rate reported as greater than 10 times the actual rate (reported as 2%)	• Descriptive Statistics
Simon et al., 2018		• Aware of indications: 88.6% “yes”, 11.4% “no” • Offer surgical option to eligible patients: ○ 0–25% of eligible patients: 32.7% ○ 26–50% of eligible patients: 23.1% ○ 51–75% of eligible patients: 17.3% ○ 76–100% of eligible patients: 26.9%		• Descriptive Statistics
Conaty et al., 2020		• Familiar with NIH eligibility criteria: 46.7% strongly agree/agree, 35.3% strongly disagree/disagree	• Comfortable informing patients about various BSY options: 51% PCPs strongly agree/agree	• Descriptive statistics • 5-point Likert Scale
El-Beheiry et al., 2020		• Use NIH criteria for referral: 26% agree, 74% deny		• Descriptive Statistics
Elliott et al., 2020		• “I have good knowledge of the criteria for referral” ○ Endocrinologists: 68% strongly agree/agree • 6–13% of otorhinolaryngologists, obstetricians/gynecologists, and orthopedic surgeons strongly agree/agree		• Descriptive Statistics • 5-point Likert Scale
Lopez et al., 2020			• Correctly identified mortality rate of RYGB: 53.7%	• Descriptive statistics
Egerer et al., 2021	• Calculate every patients BMI: 38% • Calculate BMI only if patient is noticeably overweight: 53%	• Knowledge of eligibility criteria: 65.2% familiar • Mean knowledge of eligibility criteria (1 = unfamiliar, 5 = familiar): ○ Normal weight PCP (3.64 ± 1.3), overweight/obese PCP (3.85 ± 1.0) (*p* = 0.454) ○ Male PCP (3.76 ± 1.2), female PCP (3.65 ± 1.2) (*p* = 0.445) ○ Younger PCP (3.16 ± 1.3), older PCP (3.86 ± 1.1) (*p* = 0.005)	• Knowledge of different procedure options: 86.3% familiar • Mean knowledge (1 = no knowledge, 5 = high knowledge): ○ Normal weight PCP (4.41 ± 0.8), overweight/obese PCP (4.59 ± 0.7) (*p* = 0.185) ○ Male PCP (4.58 ± 0.7), female PCP (4.33 ± 0.9) (*p* = 0.036) ○ Younger PCP (4.39 ± 0.8), older PCP (4.55 ± 0.7) (*p* = 0.305)	• Descriptive Statistics • Two-sample t-test • Mann–Whitney *U* test
Memarian et al., 2021		• Have “good knowledge” of referral criteria: 73% strongly agree/agree • 2 items on BMI criteria correctly answered: 55% strongly agree/agree		• Descriptive statistics • 5-point Likert Scale
Özgüc et al., 2021	• Never: 2.2% • Rarely: 19%; • Sometimes: 47.1% • Frequently: 28.2% • Always: 3.5%	• Correctly identified obese BMI range: 25 < BMI < 29 (93.5%), BMI > 30 (82.8%) • Patients with BMI > 40 kg/m^2^ should be referred: 72.37% agree ○ 56.3% agree, 16.1% strongly agree • Patients with BMI 35–40 kg/m^2^ and comorbidities should be referred: 53.3% agree ○ 42.3% agree, 11% strongly agree • Patients with BMI 35–40 kg/m^2^ and uncontrolled diabetes should be referred: 35.9% agree ○ 30.1% agree, 5.7% strongly agree		• Descriptive statistics • 5-point Likert Scale
Zevin et al., 2021		• Unfamiliar with eligibility guidelines for patients with class II/III obesity and T2D: 53.3% • Have “good” knowledge of the referral criteria: All PCPs (68.9%) ○ Male (63.3%), female (36.8%) (*p* = 0.018)		• Descriptive statistics • Independent sample *t*-tests
Alenezi et al., 2022			• Level of training and education level not significantly associated with higher reported knowledge	• Descriptive Statistics
Carrasco et al., 2022		• Willing to refer patient with BMI 38 kg/m^2^, several obesity-related comorbidities, family history of cardiovascular mortality: 43%		• Descriptive statistics
Holmes et al., 2022		• Correctly identified regional BMI cutoffs for BSY: 67.0%		• Descriptive statistics
Ouni et al., 2022		• Familiarity with NIH eligibility criteria: 31.5% familiar, 45.4% somewhat familiar, 23.1% unfamiliar • Knowledge of indications for EBTs: 75.4% unfamiliar, 6.2% familiar	• Awareness of EBTs for weight loss: 52.3% unaware • Interest in further education regarding therapeutic options for patients with obesity: 84.6% PCPs	• Descriptive statistics
Zawadzka et al., 2022		• Correctly identify eligibility guidelines: 35.9% of all physicians, 32.4% of diabetologists, 40% of non-diabetologists • Correctly identify criteria that indicate postponing a scheduled BSY procedure: 45.3% diabetologists, 31.3% non-diabetologists (*p* = 0.02)	• Have knowledge about perioperative mortality: 85.3% diabetologists, 56.6% non-diabetologists (*p* = 0.01) • Interest in broadening knowledge: 92.2% of physicians	• Chi-squared test
Mojkowska et al., 2023		• Percentage of correct answers to questions related to knowledge: ○ BMI reference values: 89% ○ Indications: 51% • HCPs self-assessment of knowledge of obesity was negatively correlated with actual level of knowledge	• Percentage of correct answers to questions related to knowledge about obesity: ○ Goals of surgical treatment of obesity: 66% ○ Indications: 51% • Respondents with prior training related to obesity had a lower regard of their knowledge of diagnosis and treatment (*p* = 0.008) • Respondents with prior training on obesity answered more questions correctly (*p* = 0.026) • Providers who work in hospitals had higher knowledge than providers who work in outpatient centers (*p* = 0.009) • Low level of knowledge was more often present in respondents < 29 years old than respondents > 30 years old (*p* = 0.03) • Older respondents knew regulations on reimbursements more often (*p* = 0.04)	• Pearson’s chi-squared test • Mann–Whitney *U* Test

Abbreviations: *AACE* American Academy of Clinical Endocrinology, *ASMBS* American Society for Metabolic and Bariatric Surgery, *BARI* bariatric medicine, *BS* bariatric surgeon, *BSY* bariatric surgery, *CARD* cardiologists, *ENDO* endocrinologists, *FP* family practitioners, *ICSI* Institute for Clinical Systems Improvement, *IM* internal medicine, *LAGB* laparoscopic adjustable gastric banding, *LRYGB* laparoscopic RYGB, *LSG* laparoscopic sleeve gastrectomy, *NHLBI* National Heart, Lung, and Blood Institute, *NIH* National Institute of Health, *OBGYN* obstetrics/gynecology, *PCP* primary care physician, *RYGB* Roux-en-Y gastric bypass, *TOS* The Obesity Society, *USPSTF* U.S. Preventative Services Task Force

**Table 3:** Perceptions of bariatric surgery safety and efficacy among providers

Author	General impressions	Tool for weight loss/comorbidity resolution	Safety/risks	Long-term efficacy	Statistical test
Thuan et al., 2005	• BSY “should be restricted to patients who have not responded to other treatment methods after at least 1 year of follow-up:” 87% agree or strongly agree	• “Surgery must be considered to treat obesity only in exceptional cases:” 55% strongly agree, 34% agree • “Surgery is the only possibility for obese patients to significantly reduce their weight and maintain that loss:” 26% strongly disagree, 47% disagree, 12% agree, 5% strongly agree			• Descriptive statistics
Avidor et al., 2007	• Top respondent-rated advantages: ○ Improved patient commitment (40.3%) ○ Improved self-esteem (4.3%)	• Top respondent-rated advantages: ○ Comorbidities/health/diet/reduced mortality/long term results/less medication (26.9%) ○ Improved quality of life (11.6%) ○ Immediate results (10.6%) ○ Alternative to diet, exercise, and medication (9.2%)	• The top-rated disadvantages: ○ Surgical risk/complications/morbidity/anesthesia/infections (60.1%) ○ Long-term complications/side effects/weight regain/dumping (24.8%) ○ No psychological or behavioral lifestyle modifications/long-term maintenance (9.2%) ○ Mortality rate/risk of death (4.9%)	• Long-term outcome success: ○ Achieved and maintained %EWL ○ Improved overall health ○ Reduction or resolution of comorbidities: ▪ T2D ▪ Hypertension ▪ Obstructive sleep apnea ▪ Hyperlipidemia ▪ Degenerative joint disease	• Descriptive statistics
Sansone et al., 2007	• GBS undermines other weight management methods: 34.3% agree • Saves society money in the long-run: 63.6% agree • Should be covered by insurance: 90.8% agree • Is overutilized in today’s medical community: 62.6% agree	• GBS is the only effective means of treating morbid obesity: 77.8% disagree	• GBS patients seem to have a high frequency of post-surgical complications: 69.4% agree		• Descriptive statistics
Balduf et al., 2008			• Agree benefits outweigh the risks: Referring (84.2%), non-referring (52.3%), *p* < 0.001		• Chi-squared test or Student’s *t*-test
Salinas et al., 2011		• RYGB is effective in helping patients lose weight: 37.2% agree ○ 39.2% FP, 35.2% IM • LAGB is effective in helping obese patients lose weight: 32.8% agree ○ 31.1% GP, 34.5% IM • More likely to refer for BSY if they believed it was effective than if they did not believe it was effective ○ RYGB: *p* < 0.019 ○ LAGB: *p* = 0.005	• RYGB for weight loss is safe: 4.1% agree ○ 4.8% FP, 3.5% IM • LAGB for weight loss is safe: 11.1% agree ○ 10.9% FP, 11.3% IM • More likely to refer for BSY if they believed it was safe than if they did not believe it was safe ○ RYGB: *p* = 0.001 ○ LAGB: *p* < 0.001		• Descriptive statistics
Sarwer et al., 2012	• Positive impressions: 80%	• Have positive impressions as T2D treatment: 67% • Willing to randomly assign obese patients with T2D to BSY: 80% • Believe obese patients will have significant diabetes improvement with: ○ Gastric bypass: 68% ○ Lap band surgery: 58% ○ Sleeve gastrectomy surgery: 42%			• Descriptive statistics
Claridge et al., 2014	• “Does not address the root causes of obesity” • Can be “life-changing for patients who receive it”		• Portrayed as a “drastic intervention with a high level of risk and morbidity”		• Qualitative
Kim et al., 2015	• GPs believe BSY is rarely of value with pessimism about how successful BSY is • Viewed as a “last resort” • Is successful where “major weight loss is required”	• A number of GPs recognized the value “especially for patients with comorbidities”		• Value is its contribution to “long-term maintenance of major weight loss”	• Qualitative
Stanford et al., 2015	• Strongly agree/agree BSY is useful: ○ Previous obesity related training (92.7%) ○ No obesity related training (100%)		• Strongly agree/agree BSY is safe: ○ Previous obesity related training (75.6%) ○ No obesity related training (75.9%)		• Descriptive statistics • Chi-square analyses and Fisher’s exact tests
Tork et al., 2015			• Benefits are worth the risks: 50% disagree, 12% strongly disagree ○ Referring PCP: 66% disagree ○ Non-referring PCP: 53% disagree • Not familiar with the risks and benefits: 47% disagree, 36% strongly disagree	• BSY only effective long-term treatment for weight loss: 68% strongly disagree/disagree ○ Referring PCP: 64% disagree ○ Non-referring PCP: 75% disagree • Treatment and long-term management after surgical intervention are often ineffective: 63% strongly disagree/disagree	• Descriptive statistics • 5-point Likert Scale
Auspitz et al., 2016	• Would refer a family member or friend for GBS: All FPs (81.9%) ○ Previously referred (85.4%), No history of referral (55.6%), (*p* = 0.0002) • Agree with NIH criteria: 60.1%	• Supportive of metabolic surgery for patients with diabetes and BMI < 35 kg/${\mathrm{m}}^{2}$: All FPs (48.1%) ○ Previously referred (49.3%), Non-referring (38.9%) (*p* = 0.46)		• Results in sustained weight loss: All FPs (70.7% agree), previously referred (88.7%), Non-referring (93.8%) (*p* = 0.551) • Reasons for non-referral: Limited benefits (9.4%)	• Descriptive statistics • Chi-square or Fisher’s exact tests
Funk et al., 2016			• PCPs were concerned about safety and risk of complications ○ Including: poor quality of life, reoperations, mortality	• Most PCPs believed BSY was effective in the short-term • Most PCPs expressed concern that long-term failures were common (weight regain, excessive WL)	• Qualitative
Hirpara et al., 2016	• Would refer a family member: All Surgeons (84.4%) ○ BS (95%), non-BS (81.7%) (*p* = 0.143) • Supportive of “metabolic surgery:” All Surgeons (42.5%) ○ BS (80%), non-BS (65.4%) (*p* = 0.26)			• Results in sustained weight loss for most patients: All surgeons (83.2%) ○ BS (100%), non-BS (79%) (*p* < 0.001)	• Descriptive statistics • Pearson chi-square test
Jung et al., 2016	• An easy way out: 17.59% totally disagree, 33.67% neutral, 7.54% totally agree	• Useful to reduce body weight: 56.4% agree			• Linear regression • Logistic regression
Major et al., 2016		• May help in the treatment of metabolic syndrome: 96.6%			• Descriptive statistics
Stolberg et al., 2017		• BSY as the primary future treatment of severe obesity: ○ 9% strongly agree/agree ○ 50% strongly disagree/disagree ○ 40% neither agree nor disagree	• Operative complications caused reluctance in referring: ○ 28% strongly agree/agree ○ 39% strongly disagree/disagree ○ 32% neither agree nor disagree • Postoperative surgical complications caused reluctance in referring: ○ 43% strongly agree/agree ○ 39% strongly disagree/disagree ○ 28% neither agree nor disagree • Postoperative medical complications caused reluctance in referring: ○ 40% strongly agree/agree ○ 30% strongly disagree/disagree ○ 29% neither agree nor disagree • Mental adverse reactions caused reluctance in referring: ○ 15% strongly agree/agree ○ 45% strongly disagree/disagree ○ 38% neither agree nor disagree		• Descriptive statistics • 5-point Likert Scale
Falvo et al., 2018		• BSY is an effective treatment for obesity: 65.1% agree, 9.3% strongly agree	• BSY is a safe treatment for obesity: 51.2% agree, 4.7% strongly agree		• Descriptive statistics • 5-point Likert Scale
Martini et al., 2018		• Effective in inducing T2D improvement/remission: 28.5% aware ○ 25.7% no training vs 21.4% university training vs 36% post-university education	• Overestimate post-op malnutrition rates: 86.7% • Overestimate post-op mortality rate: 26%	• Most effective bariatric procedure: ○ Gastric banding: 2.8% ○ Sleeve gastrectomy: 46.9% ○ Gastric bypass: 50.4%	• Descriptive Statistics
McGlone et al., 2018	• Objective is to improve quality of life: 97% PCPs agree	• Objective is to improve comorbidities: 100% PCPs agree	• Benefits outweigh risks for patients with obesity: 69% PCPs agree ○ 50% PCPs with ≤ 8 years of experience, 76% PCPs with > 8 years of experience, *p* = 0.13		• Descriptive statistics • Pearson chi-square test
Simon et al., 2018			• Reason for not offering: ○ Concern of adverse events: 31.9% ○ BSY would be risky since patient has many comorbidities: 57.4%	• Reason for not offering: ○ Not well aware of long-term outcomes: 16.0%	• Descriptive statistics
Conaty et al., 2020	• Is a valuable tool: 82.7% strongly agree/agree	• BMI > 40 is a greater risk than undergoing BSY: 86% strongly agree/agree	• Surgical complications/side effects as a barrier to referral: 21.48% agree	• Is an efficacious treatment: 87% of all respondents strongly agree/agree • Ineffective postoperative weight loss barrier to referral: 18.52%	• Descriptive statistics • 5-point Likert Scale
El-Beheiry et al., 2020		• PCP average estimation of co-morbidity resolution with BSY: ○ T2D (50.7% ± 23.4%) ○ Hypertension (47.3% ± 21%) ○ Dyslipidemia (43.8% ± 22.7%) ○ Obstructive sleep apnea (52.8% ± 22.3%) ○ Osteoarthritis (39.2% ± 22.4%)			• Descriptive statistics
Elliott et al., 2020		• Agree with referral on physician's initiative due to comorbidities: ○ T2D: 40% strongly agree/agree ○ Sleep apnea: 10% strongly agree/agree ○ PCOS: 10% strongly agree/agree Arthrosis: 11% strongly agree/agree	• Commonly cited concerns of referring: ○ Perioperative complications: 10–17% of providers ○ Postoperative complications: 6–28% of providers • Endocrinologists were the most concerned about referral (complications and lack of long-term follow-up)		• Descriptive Statistics • 5-point Likert Scale
Lopez et al., 2020			• Obstacles to referral: fear of complications or mortality (2.4%) • Surgery is too invasive/high risk: 12.2% agree • Risks outweigh the benefits: 43.9% agree		• Descriptive statistics
Memarian et al., 2021	• Positive attitude: 48%	• Strongly agree/agree about positive effects on: ○ T2D (90%) ○ Hypertension (82%) ○ Hyperlipidemia (65%) ○ Sleep apnea (88%) ○ GERD (62%) ○ PCOS (43%) ○ Female infertility (57%) Joint pain (75%)	• Advantages outweigh risks: 46% • Not concerned about risks: 42% • Concerned about postoperative medical complications: 50% • Concerned about postoperative surgical complications: 51% • Concerned about psychiatric side effects: 46%		• Descriptive statistics • 5-point Likert Scale
Özgüc et al., 2021	• Would refer a first-degree relative: 9.7% strongly agree, 48% agree • Disapproval in changes of physiology and anatomy: 10.8%	• Would recommend for obese patients unsuccessful in losing weight with a comprehensive diet and exercise program: 37.6%	• Highly risky: 56.4% agree	• Provides the longest and largest amount of weight loss in morbidly obese patients: 17.9% strongly agree, 54.9% agree • Not effective: 5.7%	• Descriptive statistics • 5-point Likert Scale
Sbraccia et al., 2021	• Believe there are good options available for BSY: 58% agree • Consider BSY during discussions about weight management: 10% HCPs	• Recommend BSY as an effective long-term weight management recommendation: 37% HCPs			• Descriptive statistics
Zevin et al., 2021	• Usually successful in helping patient with obesity without BSY: 87.1% disagree	• Primary treatments for class II/III obesity should be based on BSY with behavioral and dietary modifications: 43.4% agree	• Concerns about post-operative surgical complications: 54.1% • Concerns about risk: 43.5% • Concerns about postoperative medical complications: 32.9% • Concerns about psychiatric side effects: 7.3%		• Descriptive statistics • 5-point Likert Scale
Alenezi et al. 2022	• Knowledge significantly associated with attitudes (*p* < 0.001) • Education level and higher level of training significantly associated with more positive attitude (*p* = 0.005, *p* = 0.012) • Have a positive attitude: 40% PCPs		• Concerned with risk for postoperative complications: 38.6% agree, 9.3% disagree • Concerned about risks: 24.3% strongly agree, 35.7% agree	• “Long-term consequences are not completely known”: 32.1% agree, 30% neutral	• ANOVA • Independent two-tailed t-test
Murtha et al., 2022	• Providers believed patients perceived BSY as a last resort	• Not perceived as necessary until obesity or comorbidities were “life threatening” or greatly impeding physical capabilities • Weight reaching “tipping point” is motivational factor			• Descriptive statistics
Ouni et al. 2022		• Effective treatment for weight loss and improving metabolic disease: 42.3% strongly agree, 50.8% agree, 6.9% neutral • EBTs are an effective treatment option for metabolic disease: 46.2% agree, 11.5% strongly agree, 40% neutral			• Descriptive statistics
Zawadzka et al., 2022		• Effective treatment for metabolic syndrome: 90.6% agree • More effective for glycemic control than an intensive conservative treatment: 84.4% agree			• Descriptive statistics

Abbreviations: *BARI* bariatric medicine, *BS* bariatric surgeon, *BSY* bariatric surgery, *CARD* cardiologists, *EBT* endoscopic bariatric therapy, *ENDO* endocrinologists, *EWL* excessive weight loss, *FP* family practitioners, *GBS* gastric bypass surgery, *GERD* gastroesophageal reflux disease, *GP* general practitioner, *HCP* healthcare professional, *IM* internal medicine, *LAGB* laparoscopic adjustable gastric banding, *OBGYN* obstetrics/gynecology, *PCOS* polycystic ovary syndrome, *PCP* primary care physician, *RD* registered dietician, *RYGB* Roux-en-Y gastric bypass, *T2D* type 2 diabetes, *WLS* weight loss surgery

**Table 4:** Factors associated with comfort in initiating conversations about bariatric surgery and managing eligible patients

Author	Initiating discussions and providing referrals	Providing postoperative management and follow-up care	Statistical test
Avidor et al., 2007	• Initiate conversation: BARI (26.1%), OBGYN (11.9%), IM (8.8%), ENDO (17.9%), CARD (8.0%), FM (21.9%), All Specialties (15.4%) • Top reasons for not referring eligible patients: lack of acquaintance with local bariatric surgeons (37.4%), perception that patient lacked interest (23.6%) • All specialists who had previously referred patients for bariatric surgery were more familiar with local bariatric surgeons than providers who had not previously referred		• Descriptive statistics
Balduf et al., 2008		• Reported competence in addressing medical complications: Previously referred (54.2%), non-referring (15.4%) (*p* < 0.001)	• Chi-squared test or Student’s *t*-test
Ferrante et al., 2009	• Higher knowledge associated with increased frequency of recommendations: *p* < 0.0001 • Greater percentage of patients with obesity seen in practice associated with decreased likelihood of recommending BSY: OR 0.38, *p* = 0.0002		• Odds ratio • Chi-squared test
Salinas et al., 2011	• Very confident discussing LAGB: 34.2% agree ○ 34.7% FP, 33.8% IM • Very confident discussing Roux-en-Y gastric bypass: 32.1% agree ○ 31.3% FP, 32.9% IM	• Confident managing patients after LAGB: 14.4% agree ○ 13.5% FP, 15.4% IM	• Descriptive statistics
Glauser et al., 2015	• “Very significant” barriers in communicating with patients who are obese ○ Lack of Training on how to discuss obesity: 13% bariatricians, 14% cardiologists, 11% endocrinologists, 11% PCPs ○ Lack of resources to refer overweight and obese patients to: 24% cardiologists, 33% endocrinologists, 29% PCPs ○ Low likelihood of succeeding in helping patients achieve or maintain healthy weight: 33% cardiologists, 41% endocrinologists, 32% PCPs		• Descriptive statistics
Kim et al., 2015	• GPs decision to refer patients strongly influenced by patients’ expectation or request • GPs that refer patients for BSY were often influenced by “positive feedback from patients”		• Qualitative
Stanford et al., 2015	• Barriers to evaluating/managing patients: ○ Prior obesity training: Insufficient time (61%), not being part of professional role (63%), inadequate training (71%), fear of offending patient (100%), too difficult for patients to change (59%), lack of effective tools and information to give to patients (65%), long wait times for referrals to obesity medicine specialists (54%) ○ No obesity training: Inadequate reimbursement (75%), lack of adequate referral services (57%), patients being generally not interested in improving their weight status (58%), lack of effective treatment options (52%) • Statistically significant factor associated with physician confidence in treating obesity: prior obesity training	• Statistically significant factors associated with physician confidence in treating obesity: younger physician age, physician’s own BMI being higher	• Descriptive statistics • Chi-square analyses and Fisher’s exact tests
Tork et al., 2015	• Do not feel competent to discuss BSY as a treatment option for morbidly obese patients: 70% PCPs strongly disagree/disagree	• Not comfortable managing postoperative patients: 45% PCPs strongly disagree/disagree	• Descriptive statistics • 5-point Likert Scale
Auspitz et al., 2016	• Initiate conversation: 73.1% FPs • Reasons for non-referral: Disagree with the procedure (7.6%), concerned with follow-up care (24.5%), discomfort within own practice to manage patients with obesity (7.6%)	• “I feel comfortable providing care to patients who have received BSY:” All FPs (46.5%) ○ Previously referred (64.2%), non-referring (26.7%) (*p* = 0.005)	• Descriptive statistics • Chi-squared or Fisher’s exact test
Funk et al., 2016	• Requirement of PCPs to address postoperative issues contributed to hesitation to refer patients for BSY		• Qualitative
Hirpara et al., 2016	• Comfortable explaining procedure: All Surgeons (37%) ○ BS (100%), non-BS (50%) (*p* < 0.001) • Initiate conversation: All Surgeons (59.6%) ○ BS (94.7%), non-BS (51.3%) (*p* = 0.001)	• Managed > 20 patients with previous BSY (past 12 months): All Surgeons (16.8%) ○ BS (70%), non-BS (3.7%) (*p* < 0.001) • Managed complications (past 12 months): All Surgeons (82.2%) ○ BS (100%), non-BS (77.7%) (*p* < 0.001) • Confident managing common early complications: All Surgeons (38.6%) ○ BS (95%), non-BS (24.7%) (*p* < 0.001) • Confident managing common late complications: All Surgeons (41.6%) ○ BS (95%), non-BS (28.4%) (*p* < 0.001) • Able to transfer patients effectively: All Surgeons (32.7%) ○ BS (42.1%), non-BS (34.6%) (*p* = 0.18)	• Descriptive statistics • Pearson chi-square test
Jung et al., 2016	• Factors related to frequency of recommendation: ○ Perceived expertise (*p* < 0.001) ○ Rating of BSY as “useful” (*p* < 0.001) ○ Attitude towards BSY as an “easy way out” (*p* < 0.001)		• Linear regression • Logistic regression
Major et al., 2016	• Have previously referred a patient: 72.2% PCPs		• Descriptive statistics
Stolberg et al., 2017	• Had extensive experience: 45% disagree/strongly disagree • Reluctant to refer patients due to negative experiences: ~ 30% • Discuss referral on their own initiative with a patient with severe obesity and type-2 diabetes: 22% PCPs agree/strongly agree		• Descriptive statistics • 5-point Likert Scale
Zacharoulis et al., 2017	• Highest rates of non-referral: cardiologists (90.2%), endocrinologists (90.0%) • Lowest rates of non-referral: general surgeons (58.3%)		• Descriptive statistics
Falvo et al., 2018	• Initiate conversation: 76.6% PCPs frequently, 17.8% PCPs always		• Descriptive statistics
Martini et al., 2018	• Open discussion: 36.1% GPs • Refer eligible patients: 56.6% ○ 46.6% post-university obesity education, 19.6% university education (*p* < 0.05) ○ 46.2% with BSY recommendations knowledge, 31.3% without knowledge (*p* < 0.05)	• Available for post-operative follow-up: 83.7% • Want education in post-operative follow-up: 86.1%	• Descriptive statistics • Pearson chi-square test
McGlone et al., 2018		• Confident with providing long-term postoperative care: 34% ○ 30% PCPs with ≤ 8 years of experience, 36% PCPs with > 8 years of experience, *p* = 0.99 • Feels well-supported in managing postoperative medical problems: 17% ○ 10% PCPs with ≤ 8 years of experience, 20% PCPs with > 8 years of experience, *p* = 0.63 • Feels well-supported managing postoperative surgical problems: 8% ○ 10% PCPs with ≤ 8 years of experience, 8% PCPs with > 8 years of experience, *p* = 0.99	• Pearson chi-square test • Descriptive statistics
Simon et al., 2018	• In patients with BMI 30–39 kg/m^2^, 40.7% discuss BSY • In patients with BMI ≥ 40 kg/m^2^, 76.4% discuss BSY • Reason for not offering: ○ Limited experience on when to offer BSY: 26.6%		• Descriptive statistics
Conaty et al., 2020		• Comfortable managing long-term care of postoperative patient: 59.5% PCPs strongly agree/agree	• Descriptive statistics • 5-point Likert Scale
El-Beheiry et al., 2020	• Comfortable counseling patients: All FPs (45.4%) ○ Referring (56.8%), non-referring (17.1%), *p* < 0.001	• Comfortable with post-operative care: 9.4% strongly agree, 50.4% agree ○ Referring (67.4%), non-referring (38.2%), *p* = 0.004	• Descriptive statistics • Student’s *t*-test
Lopez et al., 2020	• Obstacles to referral among PCPs: ○ Lack of patient interest/engagement (61%) ○ Time restriction during patient visits (14.6%) ○ Prior experiences of poor patient outcomes (12.2%) ○ Feeling unclear how to refer (2.4%) • Initiate discussions regarding weight loss management greater than 50% of the time: 51.2% PCPs ○ Male provider (76.5%), Female provider (37.5%), *p* = 0.01		• Descriptive statistics • ANOVA and chi-squared tests
Egerer et al., 2021	• Average confidence in approaching obese patients concerning their weight and related risks (5 point scale) ○ Normal weight PCP (4.44 $\pm$ 0.7), Overweight PCP (4.47 $\pm$ 0.7), *p* = 0.756 ○ Male PCP (4.45 $\pm$ 0.7), Female PCP (4.46 $\pm$ 0.7), *p* = 0.947 ○ Younger PCP (4.35 $\pm$ 0.8), Older PCP (4.48 $\pm$ 0.7), *p* = 0.538 • Confidence in educating obese patients regarding their obesity (5 point scale) ○ Normal weight PCP (4.22 $\pm$ 0.9), Overweight PCP (4.32 $\pm$ 0.8), *p* = 0.549 ○ Male PCP (4.30 $\pm$ 0.8), Female PCP (4.24 $\pm$ 0.9), *p* = 0.783 ○ Younger PCP (3.90 $\pm$ 1.0), Older PCP (4.38 $\pm$ 0.8), *p* = 0.009	• Average number of patients provided with follow-up care after BSY (1 = 0 patients, 5 = more than 5 patients) ○ Normal weight PCP (3.20 $\pm$ 1.3), Overweight PCP (3.53 $\pm$ 1.4), *p* = 0.089 ○ Male PCP (3.59 $\pm$ 1.4), Female PCP (3.06 $\pm$ 1.2), *p* = 0.014 ○ Younger PCP (2.77 $\pm$ 1.2), Older PCP (3.49 $\pm$ 1.2), *p* = 0.007	• Descriptive statistics • Two-sample *t*-test • Mann–Whitney *U* test
Memarian et al., 2021	• Felt competent to discuss: 59% PCPs • Initiate conversation: 7% PCPs	• Felt competent to take care of patients post-op: 63% PCPs	• Descriptive statistics
Sbraccia et al., 2021	• Correlation between PCP weight and likelihood of reviewing BSY options with patients: 58% normal weight HCPs, 44% overweight or obese HCPs • Agree that patients trust them to recommend BSY if appropriate: 62% normal weight HCPs, 48% overweight or obese HCPs		• Descriptive statistics
Zevin et al., 2021	• Percentage of PCPs who initiate conversation based on number of years in practice ○ 0–10 years (29.6%), 11–20 years (100%), 21–30 years (70%), 31 + years (76.9%) ○ 0–10 years vs 11–20 years (*p* = 0.002) ○ 0–10 years vs 21–30 years (*p* = 0.016) ○ 0–10 years vs 31 + years (*p* = 0.013) • Agree they initiate conversation: 44.8% PCPs • Correlation between PCP age and likelihood of initiating discussion (*r* = 0.363, *p* = 0.003)	• Felt competent in addressing post-op medical complications: 18.4% PCPs • Felt comfortable providing long-term follow up: 25% PCPs	• Descriptive statistics • Independent sample *t*-tests • Pearson’s correlation coefficient
Alenezi et al. 2022	• Do not feel confident discussing BSY as a treatment option for obesity: 20% PCPs agree, 42.1% PCPs disagree	• Do not feel comfortable providing postoperative management care: 28.6% agree, 27.9% disagree	• Descriptive statistics
Carrasco et al., 2022	• Greater confidence discussing BSY with patients associated with: ○ Greater knowledge (*p* = 0.002) ○ Positive attitude towards BSY (*p* = 0.008) • Obstacles to discussing weight loss management with patients with obesity ○ Doctors lack knowledge about weight management: 31% • Confident suggesting BSY: 81%		• Multiple linear regression
Holmes et al., 2022	• Most common barriers to referral: time constraints, did not consider BSY • One-third unsure of referral process and risks/benefits		• Descriptive statistics
Ouni et al., 2022	• Comfortable referring patients: 70% PCPs comfortable, 6.9% PCPs unfamiliar with referral process for weight management		• Descriptive statistics
Zawadzka et al., 2022	• Frequency of referral: diabetologists more frequently refer patients to BSY consultations compared with non-diabetologists (*p* = 0.034) • Self-estimated knowledge of provider significantly associated with greater frequency of recommending BSY to eligible patients (*p* = 0.0016)	• Have knowledge about recommended plan for outpatient postoperative follow-up: 68.8% of all physicians, 73.5% of diabetologists, 63.3% of non-diabetologists	• Chi-squared test

Abbreviations: *BARI* bariatric medicine, *BMI* Body Mass Index, *BS* bariatric surgeon, *BSY* bariatric surgery, *CARD* cardiologists, *EBT* endoscopic bariatric procedure, *ENDO* endocrinologists, *LAGB* laparoscopic adjustable gastric banding, *PCP* primary care physician, *RD* registered dietician

The quality and risk of bias of each study were independently assessed by two reviewers using the Newcastle–Ottawa Scale (NOS) for cross sectional studies. Any discordance in assigned scores between reviewers was resolved by a third reviewer. Studies are graded based on several categories, with a maximum of five points awarded for selection, two points for comparability, and three points for outcomes, resulting in a total maximum score of ten points. We noted scores of < 5, 5–6, and 7–10 to be unsatisfactory, satisfactory/good, and very good quality, respectively. The NOS score for each study is listed in Table 1.

## Results

### Overview of Studies

**Fig. 2:**
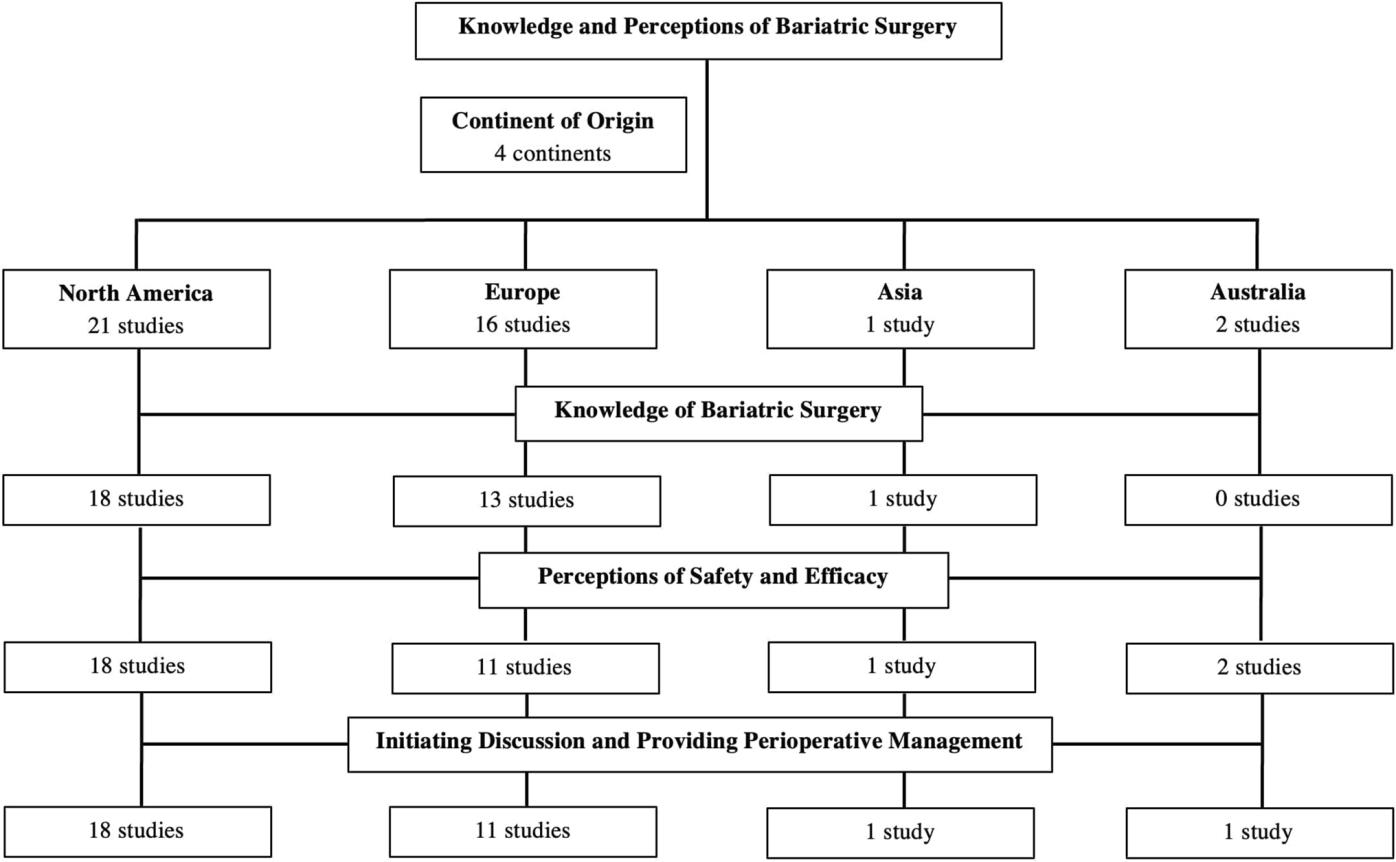
Summary of regional distribution and areas of emphasis of included studies

Forty studies were included in this review. Among these studies, 39 [11–49] were cross-sectional and 1 [50] was prospective. A total of 36 studies [11–16, 18–20, 22–24, 26–43, 45–50] utilized a survey or questionnaire while 4 were interview-based [17, 21, 25, 44]. Many solely examined perceptions of family practitioners (FPs) or primary care physicians (PCPs) [11, 15–17, 21–25, 27, 28, 30, 31, 33, 34, 36–39, 41–43, 45, 49], while some examined perceptions of providers from multiple specialties, such as surgery, endocrinology, and internal medicine [12–14, 18–20, 26, 29, 32, 35, 40, 44, 46–48, 50]. A total of 40% studies were conducted among physicians in the USA (Fig. 2). According to the NOS, 20.0% were very good studies, 50.0% were satisfactory/good, and 30.0% were unsatisfactory. A summary of study designs, sample sizes, NOS scores, and response rates is shown in Table 1.

### Eligibility

Twenty-five studies [12, 14, 16, 19, 20, 22, 23, 26, 28, 30, 32–35, 37–39, 41, 43, 45–50] assessed provider familiarity with eligibility for bariatric surgery (Table 2). Six studies specifically explored familiarity with the National Institute of Health (NIH) eligibility criteria [12, 14, 26, 33, 34, 45], while the remaining studies investigated familiarity of criteria or indications without specifying NIH as the source, by posing mock cases to providers, or evaluating utilization of different criteria accepted in their respective regions.

On average, fewer than 50% of providers reported reading or being familiar with NIH criteria. Notably, providers with prior training in bariatric or obesity medicine were found to have greater familiarity with eligibility guidelines [12, 22, 26], as were providers with a history of providing referrals for bariatric surgery [14]. In the USA, among the few studies identifying strong familiarity with surgical indications, Tork et al. (2015) found that 85% of surveyed PCPs in a private teaching hospital in Cincinnati strongly agreed or agreed that a BMI > 35 kg/m^2^ and comorbidities were an indication for a surgical referral [23]. Among physicians outside the USA, on average, a majority indicated higher familiarity with the national eligibility criteria or established indications for bariatric surgery. For instance, Major et al. (2016) and Memarian et al. (2021) found that 81.8% of surveyed PCPs in Poland and 73% of PCPs in Sweden knew the indications for a bariatric procedure or agreed that they had good knowledge of referral criteria, respectively [37, 50]. Of note, among 204 PCPs in a bariatric surgery center at a university hospital in Germany, older PCPs were found to have significantly higher mean knowledge of national eligibility criteria than younger PCPs (*p* = 0.005) [38].

### General Knowledge

Twenty-two studies [12, 14, 15, 19, 20, 22, 24–27, 29–31, 33, 36, 38, 42, 45–47, 49, 50] examined knowledge of bariatric procedures (Table 2). Nine studies specifically queried physicians regarding knowledge of differences between bariatric procedure options [12, 24–26, 29, 38, 45, 49, 50], with a majority self-reporting an average level of familiarity. A history of providing referrals or previously receiving obesity medicine-related training was associated with greater knowledge of bariatric procedures and familiarity with expected surgical outcomes. Among physicians across various specialties in Greece, below 15% of surveyed providers reported “a lot” of familiarity with each of six bariatric procedures posed to them [29]. At the time of the study, Roux-en-Y gastric bypass and laparoscopic sleeve gastrectomy were the most common bariatric procedures performed globally, yet 56.0% and 40.3% of surveyed physicians within this study reported no familiarity with Roux-en-Y gastric bypass and laparoscopic sleeve, respectively [29]. Egerer et al. (2021) found that 86.3% of surveyed PCPs in a university hospital bariatric center in Germany reported familiarity with bariatric surgery surgical options, with male PCPs self-reporting higher knowledge of bariatric procedures compared to female PCPs (*p* = 0.0036) [38]. Among multiple cohorts of providers with limited reported knowledge of bariatric surgery, an interest in broadening knowledge was frequently reported [19, 23, 24, 26, 37, 39, 45, 46, 49, 50].

### Weight Loss and Comorbidity Resolution

Twenty-seven studies [11, 12, 16, 18, 21, 23–28, 30–35, 37, 39–42, 44–46, 49, 50] examined provider impressions of bariatric surgery’s short- and long-term efficacy for weight loss and resolution of comorbidities (Table 3). A majority of studies reported positive perceptions among providers regarding the utility of bariatric surgery for sustained weight loss and improving comorbidities [12, 18, 21, 23–26, 30–35, 37, 39, 42, 45, 46, 50], of which a majority surveyed primarily PCPs. Memarian et al. (2021) found that surveyed PCPs in South Sweden strongly agreed or agreed that bariatric surgery could have a positive effect on T2DM (90%), hypertension (82%), and hyperlipidemia (65%) [37]. Among PCPs in Turkey, 17.9% strongly agreed and 54.9% agreed that bariatric surgery lead to the longest and greatest amount of weight loss in eligible patients [39]. On the other hand, a few studies noted negative perceptions, with less than half of providers from primarily multidisciplinary cohorts perceiving bariatric surgery as an efficacious option [35, 40, 44, 49]. Among healthcare providers from multiple specialties in Italy, only 37% stated they would recommend bariatric surgery to patients as an effective modality for long-term weight management [40]. A qualitative study of providers from a Veteran Affairs Medical Center in the USA reported that bariatric surgery was not perceived as necessary until obesity or its comorbidities were deemed “life threatening” [44].

### Safety

Nineteen studies [12, 14, 16, 17, 22, 23, 25, 28, 30–33, 35–37, 39, 41, 42, 49] examined provider perceptions of the safety of bariatric surgery (Table 3). Only two studies found over half of surveyed providers believed bariatric surgery was a safe treatment for obesity [22, 30]. Most of the literature reported prevalent concerns among providers from various specialties regarding physical and psychological complications. The most reported disadvantages of bariatric surgery were related to perceptions of surgical risks and postoperative complications rather than efficacy. Seven studies assessed physician knowledge of evidence-based morbidity and mortality rates of various bariatric procedures [22, 24, 26, 31, 36, 46, 49]; on average, fewer than half of surveyed providers were aware of established rates. A small number of studies assessed perceived risks of surgery versus living with obesity [14, 23, 33, 36, 37]. Some studies found an overwhelming majority of physicians agreed the benefits of surgery outweighed the risks [14] and that the risks of obesity posed greater health risks [33], while others found conflicting results [23, 36, 37]. Notably, in a sample of PCPs from a private teaching hospital in Cincinnati, 50% disagreed and 12% strongly disagreed that the benefits of bariatric surgery are worth the risks [23].

### Initiating Discussions, Providing Referrals, and Postoperative Management

Twenty-six studies [12, 15, 16, 20–24, 26–30, 32, 34, 36, 37, 40–43, 45, 46, 48–50] examined the frequency of providers initiating conversations about bariatric surgery and factors associated with providing referrals (Table 4). Across all studies, there was significantly greater reported confidence, comfort, and frequency of initiating conversations about bariatric surgery among physicians with prior training in bariatric and/or obesity medicine or greater reported knowledge of bariatric care [12, 22, 26, 27, 43, 46, 49]. There was also a well-documented relationship between a history of providing bariatric surgical referrals and greater comfort discussing bariatric surgery with patients and providing perioperative care [14, 24, 34]. Fourteen studies [14, 16, 23–26, 31, 33, 37, 38, 41, 42, 46, 49] noted that positive predictors of greater comfort included prior bariatric and/or obesity medicine training, experience with bariatric surgery, and previously providing bariatric surgical referrals. Due to small samples and a relative lack of studies, there is limited quality of evidence regarding the impact of years of clinical experience on the likelihood of discussing bariatric surgery or providing postoperative care.

The frequency of initiating discussion also varied with physician demographics. One study among surveyed PCPs in Wisconsin found that male practitioners initiated discussions with patients about weight loss management, including bariatric surgery, 76.5% of the time, while female practitioners reported doing so 37.5% of the time (*p* = 0.01) [36]. Zevin et al. (2021) noted a positive correlation between PCP age and likelihood of initiating discussions about bariatric surgery in Ontario (*r* = 0.363, *p* = 0.003) [41]. Sbraccia et al. (2021) also found that a higher proportion of PCPs of a normal weight versus PCPs who have overweight or obesity were likely to review bariatric surgery options with patients, though there was no comparative analysis conducted (58% and 44%, respectively) [40].

Additionally, eight studies found that a lack of training, perceived lack of resources, or a fear of offending the patient were commonly cited barriers to referral [22, 24, 28, 36, 48] or obstacles to discussing bariatric surgery with eligible patients [20, 22, 24, 32, 43]. Concerns regarding adverse outcomes of surgery were also one of the highest rated barriers to discussing bariatric surgery or providing referrals [28, 32, 33, 35–37, 41]. Knowledge of bariatric surgery further appeared to contribute to referral rates; a cohort of physicians in Michigan cited unawareness of long-term postoperative outcomes as a reason not to offer bariatric surgery to eligible patients [32]. There was also variation in reported barriers to referral between physicians with and without a background in bariatric training. Interestingly, a study conducted among 76 PCPs affiliated with Massachusetts General Hospital found that a fear of offending the patient and inadequate training were the most commonly cited barriers to managing bariatric surgery patients among providers with prior training, while those with no prior training most commonly cited inadequate reimbursement [22].

## Discussion

Despite its established safety and efficacy, bariatric surgery remains underutilized for the treatment of obesity. While the etiology of this underutilization is likely multifactorial, the perceptions and familiarity of healthcare providers with bariatric surgery are important factors to consider. We conducted a systematic review of the literature to assess healthcare provider familiarity with bariatric surgery, comfort with initiating discussions and perioperative management, and overall perceptions regarding its safety and efficacy. Across specialties, there was a consensus that bariatric surgery is an efficacious treatment for obesity and its associated medical conditions. However, providers often overestimated the risk profile and reported low familiarity with postoperative complication rates. Notably, prior training in obesity or bariatric medicine, greater number of years of clinical experience, and a history of providing referrals were associated with greater knowledge and greater comfort with initiating discussions about bariatric surgery and providing perioperative care. Our results highlight concerning gaps in knowledge among healthcare providers regarding the safety of bariatric surgery, and the ensuing reluctance to recommend surgical treatment for patients with severe obesity.

The role of previous obesity or bariatric training in improving provider familiarity with bariatric surgery eligibility, management, and outcomes is well-documented [12, 22, 26, 27, 43, 46, 49]. However, despite existing efforts to incorporate exposure to bariatric care into provider training, attitudes towards bariatric surgery continue to reflect concerns about surgical risks that are neither empiric nor reflective of advances towards safer, minimally invasive approaches. These concerns may be contributing to a decreased likelihood of discussing bariatric surgery with eligible patients and providing referrals for patients with severe obesity [10, 28, 32, 33, 35–37, 41, 48]. The gravity of this trend cannot be understated, considering the rising burden of the obesity epidemic and the increasingly inadequate number of non-bariatric specialists who are equipped and willing to provide high-quality bariatric management [1, 22]. In an effort to enhance provider knowledge, bariatric education that addresses the most significant deficits in knowledge should be a required component of provider education.

We recommend an expansion of the educational infrastructure in bariatric surgery and obesity medicine, with an emphasis on surgical safety and initiating the referral process for eligible patients. Barriers to the surgical treatment of severe obesity are multifold, but provider reluctance to offer surgical options due to inadequate training should be remedied at the training level. Providing bariatric surgery-specific education is essential for all specialties and levels of training, given multidisciplinary teams are at the core of managing the systemic effects of obesity [51, 52]. Therefore, bariatric training should begin prior to specialization as a part of the core medical curriculum. We expect high demand for these educational opportunities given many students and providers have reported an eagerness to learn more about bariatric surgery [19, 23, 24, 26, 37, 39, 45, 46, 49, 50]. With successful implementation, this training not only has the potential to mitigate many limitations inherent to restricting bariatric training to specialists [53] but also decrease barriers to care often experienced by patients struggling with obesity and metabolic disease.

Education and instruction on optimal strategies to approaching the conversation between providers and patients regarding bariatric surgery should also be provided in all training programs. Six studies showed provider perceptions of patients’ beliefs or a fear of offending patients impacted their willingness to discuss bariatric surgery as an option [21, 22, 33, 36, 44, 48]. Negative perceptions among providers regarding bariatric patients may also lead them to question patient motivation and ability to achieve desired weight loss postoperatively [44, 54]. Some providers reported a decreased willingness to discuss or refer eligible patients for a bariatric procedure if the patients did not show interest or demonstrated significant fear of surgery [12, 21, 28, 34, 45]. Two studies showed providers noted higher likelihood of referring the same patient if the patient initiated the discussion and expressed a strong desire to undergo the procedure [28, 49]. The preconceptions held by providers may foster distrust between the physician and patient and ultimately may impact physician counseling when discussing the possibility of a referral. This may also further exacerbate pre-existing inequities in bariatric surgery, such as racial disparities due to implicit bias and systemic racism [55, 56]. This impact of stigma on physician willingness to provide high-quality care is not new—its adverse effects have been seen in numerous other sectors, notably for patients with HIV and mental health disorders [57, 58]. However, as abundant research in these realms has pointed out, discomfort should not lessen standards of screening and care for these patients. Therefore, for the management of bariatric patients, guidance on best practices to establish rapport, conveys information comprehensibly, and respectfully encourage discourse on surgical options and outcomes may improve utilization rates and relieve the burden of this condition for affected patients.

Looking to the future, recent advances in technology may provide an avenue to bridge the gap in both provider and patient knowledge of bariatric surgery management and outcomes. Notably, there is a growing body of literature demonstrating the impressive ability of recent artificial intelligence platforms in answering clinically related questions [59, 60], including an ability to accurately and reliably answer commonly asked questions related to bariatric surgery [61]. While the literature examining the efficacy and safety of these tools in medicine is in its infancy, it has the potential to serve as an adjunct source of information for patients and providers and may facilitate physician–patient discussions regarding a bariatric surgery referral.

### Limitations

The quality of studies may have been impacted low response rates, prevalent use of self-administered surveys, and a lack of comparative analysis. There were also several studies which surveyed both patients and providers, resulting in limited extractable data for our population of interest. The NOS, though a widely recognized tool for evaluating non-randomized studies, has been critiqued for potential biases and poor inter-rater reliability, potentially contributing to misinterpretations of cross-sectional study quality [62]. Additionally, despite the use of three comprehensive databases, relevant studies published in other databases may have been inadvertently omitted. Our literature search pathway, constrained by specific keywords, may also have omitted relevant studies utilizing alternate phrasing within titles or abstracts. These limitations create avenues for future research emphasizing the importance of refined assessment tools and more robust search strategies for a comprehensive understanding of this important topic.

## Conclusion

Healthcare providers perceive bariatric surgery as an effective treatment for obesity and its comorbidities but often reported concerns regarding safety and reported low familiarity with postoperative complication rates. Gaps in education may be contributing to poor referral rates and ultimately the underutilization of bariatric surgery worldwide, all of which serve as significant barriers to best practices and standard care of the patient diagnosed with severe obesity. A history of training in bariatric or obesity medicine was associated with greater knowledge of bariatric surgery, confidence initiating discussions with patients, and providing perioperative care. Given the profound systemic effects of severe obesity on patients, combined with the rising prevalence of severe obesity, we advocate for more focused bariatric training beginning prior to specialization, with an emphasis on safety and knowledge required to provide surgical referrals. Further research investigating the effect of earlier bariatric training is required to further improve provider knowledge and increase utilization of bariatric surgery.

## Data Availability

We present no new data. All data is publicly available.

## Declarations

### Registration and Protocol

This review is not registered. A review protocol was not prepared prior to submission.
